# Robotics to Enable Older Adults to Remain Living at Home

**DOI:** 10.1155/2012/538169

**Published:** 2012-12-04

**Authors:** Alan J. Pearce, Brooke Adair, Kimberly Miller, Elizabeth Ozanne, Catherine Said, Nick Santamaria, Meg E. Morris

**Affiliations:** ^1^Cognitive and Exercise Neuroscience Unit, School of Psychology, Deakin University, Melbourne, VIC, Australia; ^2^Department of Physiotherapy, The University of Melbourne, Melbourne, VIC, Australia; ^3^Department of Social Work, The University of Melbourne, Melbourne, VIC, Australia; ^4^Department of Nursing, The University of Melbourne and Royal Melbourne Hospital, Melbourne, VIC, Australia; ^5^School of Allied Health, La Trobe University, Melbourne, VIC, Australia

## Abstract

Given the rapidly ageing population, interest is growing in robots to enable older people to remain living at home. We conducted a systematic review and critical evaluation of the scientific literature, from 1990 to the present, on the use of robots in aged care. The key research questions were as follows: (1) what is the range of robotic devices available to enable older people to remain mobile, independent, and safe? and, (2) what is the evidence demonstrating that robotic devices are effective in enabling independent living in community dwelling older people? Following database searches for relevant literature an initial yield of 161 articles was obtained. Titles and abstracts of articles were then reviewed by 2 independent people to determine suitability for inclusion. Forty-two articles met the criteria for question 1. Of these, 4 articles met the criteria for question 2. Results showed that robotics is currently available to assist older healthy people and people with disabilities to remain independent and to monitor their safety and social connectedness. Most studies were conducted in laboratories and hospital clinics. Currently limited evidence demonstrates that robots can be used to enable people to remain living at home, although this is an emerging smart technology that is rapidly evolving.

## 1. Introduction

Throughout the world rapid population ageing is occurring, with a large proportion of older adults preferring to stay living at home [1]. Most older people experience one to three chronic diseases [2] and, in very advanced age, frailty, disability, and social isolation are common. At the same time there are increasing demands on health service providers due to the low availability of home and community services, low uptake of e-health and smart technologies by healthcare professionals, and an ageing health workforce [3]. Although many older people express their desire to stay in the familiar social environment of their own home [4], many cannot do so due to impairments, immobility and social isolation. Many older people who live at home are at high risk of falls and injuries and report difficulty accessing health care services when they need them [5]. 

As previously discussed by Rowe and Kahn [6] the definition of successful aging requires three pillars. Firstly, there is a low probably of disease and/or disability from disease; secondly a high cognitive and physical functioning capacity; and three, the combination of the first two with an active engagement in life. In affecting successful aging, particularly with the nexus to an active engagement in life, there is a need for development of, and access to, smart technologies to monitor and maintain health and wellbeing, as well as to link older people with communities and healthcare professionals. One area where technology is rapidly advancing is robotics. Robots are now available that provide services such as home cleaning, appliance operation, and safety monitoring. These “service robots” can be excellent for monitoring, surveillance, and basic tasks of everyday living yet they lack artificial intelligence. Morris et al. [3] argued the need for “smart” robotic technologies that not only respond to an individual's needs, but can also learn and modify their behaviour based upon their owner's requirements. This is particularly the case for older individuals who would need to interact with their robot to maintain mobility, health, safety, and social connectedness. 

Service robots currently include commercialised domestic robots, such as self-navigating vacuum cleaners and mops, known as Roomba and Scooba respectively [7]. Service robots also include “pet” or sociable robots, such as the Aibo robotic pet dog, Paro the robotic pet seal, and similar robotic animals that use “pet therapy” to assist older people to maintain mobility, and to keep active [8]. Service robots have also been developed for hospital settings. One example of this is the iWARD project in Germany [9] where modular designed robots have been adapted for different roles for independent living, health, and safety. They can also act in a team to service the needs of medical and other health professional staff such as for remote consultations and communication between staff in different wards. 

The literature reveals some misconceptions about the potential for robotic interaction with humans. For example, popular opinion holds that robotic technologies are only applied to individuals when they are disabled [10]. However, there is a small yet increasing awareness that robotic technologies can also complement current health care service provision by monitoring older people within their home environment [11] and assisting them to mobilise safely and prevent falls [12]. Narrative literature reviews on the role of robotics in health care [8, 11–13] or social assistance robots [14] have previously been completed mainly speculating about the future of robotics in health. The aim of this systematic review was to identify specific evidence-based research answering questions to address the potential of robotic technologies to monitor older individuals' health and wellbeing and to assist with activities of daily living. Another aim was to review the extent of robotic technologies currently tested and used in the home environment for older individuals.

## 2. Methods

We identified two key questions for the systematic review of the literature addressing robotics and ageing in the home environment.

1. What is the range of robotic devices available to enable older people to remain mobile, independent and safe?
2. What is the evidence demonstrating that robotic devices are effective in enabling independent living in community dwelling older people?

Where possible, in each database, searches for all topics were limited to peer-reviewed publications between January 1990–February 2012, published in English. We included human participants aged 45 years and older, as it is generally accepted that many chronic conditions may have their onset from approximately this age onwards. This broader definition of older individuals' was adopted by the authors, defined by MESH heading definitions of “middle-aged” 45–64 years, “aged” (65–79 years) and “aged 80+ years” with the understanding that “older individuals” were a heterogeneous group. The authors also accepted the definition of a “home” setting as the individual's place of residence [15]. This included establishments providing residence and care for special needs, such as retirement villages and aged care facilities providing low care services, service integrated housing, and supported accommodation. 

To answer question 1, randomised controlled studies, quasi-experimental studies, and comparative studies with and without concurrent controls, case-series and feasibility studies, systematic and general review articles, and government reports (where relevant to topic area) were included to identify available technologies. The following publications were excluded from the paper: narrative reviews, descriptive or narrative papers without presentation of data, limited-review conference proceedings and abstracts, higher degree research theses (PhD/Masters), undergraduate research theses (Honours) and books.

To answer question 2, data extraction and quality assessments were predominantly performed on studies that met the criteria for question 1, however these studies were required to demonstrate that testing and/or data collection had been completed in a home (or simulated) environment.

Data base searches were limited to studies assessing humans and those published in English and included: Web of Science, Science Direct, MEDLINE, PSYCHINFO, SCOPUS, CINAHL, expanded version of the cumulative index to nursing and allied health by *EBSCO*, Australasian Medical Index, National Library for Health, Rehabilitation Research (USA), and TROVE.

Two independent, trained reviewers evaluated the title and abstracts of the yield articles against the decision rules inclusion criteria. The title of each article was scanned and the two reviewers independently excluded articles not related to the topic. The full texts of the articles were then obtained for data extraction, categorized according to National Health and Medical Research Council (NHMRC) guidelines on levels of evidence [16], and the quality of each article was assessed using the Downs and Black [52] quality appraisal tool. Downs and Black was specifically selected to assess the articles as it can be used for both experimental and quasi-experimental research designs. Two independent reviewers conducted data extraction and quality assessment for each article. Lack of agreement about inclusion of articles, data extracted, or grading against quality criteria was reconciled by mutual agreement. 

## 3. Results

**Figure 1:**
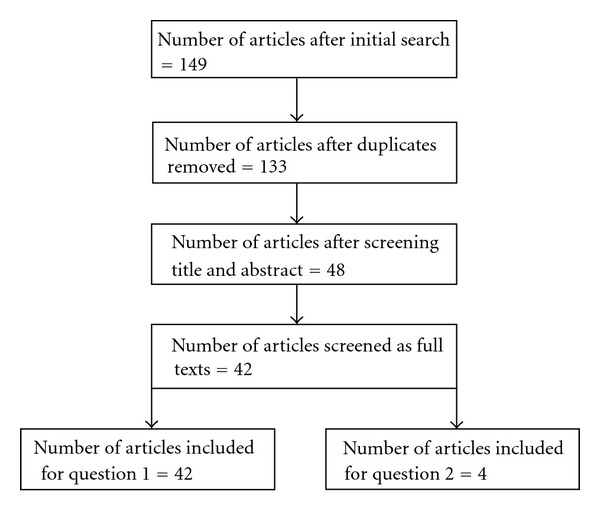
Yield of articles for the robotics literature.

Figure 1 illustrates the breakdown of articles following the predetermined inclusion and exclusion criteria. The major reason for exclusion was that articles were descriptive and did not contain data providing evidence of effectiveness, feasibility, or validity.  Table 1 shows the studies that have provided evidence of technologies assisting older people. 

The yield of articles in response to question 1 showed that robotic technology is currently available to assist older people and people with physical disabilities. These were not “smart” robots per se, with no artificial intelligence interface, and the majority of these articles were lower limb “exoskeleton” technologies. Robotic exoskeletons are fitted to the outside of the limbs, rather than being internally fixed using surgical methods and supplies at the energy (or part of the energy) for limb movement. The “Lokomat” was the most widely tested robotic exoskeleton for the lower limbs [25, 27, 29, 33, 37, 48, 49] to trial its suitability as a supportive structure for walking. Other technologies to assist with walking and mobility included robotic walkers and robotic guidance systems [30, 38]. These systems, such as the “Guido”, are extensions on the non-motorised walker frames, where the individual can control the speed of locomotion but also obtain environmental feedback, via sensors, to assist in obstacle avoidance and in navigating through doorways. 

Upper limb technologies included both upper limb exoskeleton systems to guide arm movements and haptic visuomotor feedback systems to assist in compensation for disorders of sensation and visual impairment. The “MIT-MANUS”, a visuomotor guidance system, was the most utilised of the upper limb robotic systems, particularly for people who were recovering from stroke [17, 19, 21, 24, 35]

Table 2 shows the articles that met the inclusion criteria for question 2. To date, four investigations have tested robots within a home, residential care setting, or simulated home environment. Generally, these studies demonstrated that robots are able to help older people with mobility issues around the home environment. However, this data presented was only low to moderate in terms of their level of evidence and research quality [52]. Shimada et al. [40] investigated the effectiveness of a lower limb exoskeleton device using a pre-post single group design in older healthy females within an independent home living facility. Unlike other lower limb robots, such as the Lokomat which is a relatively large driven gait orthosis that automates locomotion therapy, the exoskeleton technology in this study was smaller and more compact. This study reported improvements in walking speed and reduced energy expenditure (due to fitness gains) following 3 months of 2 sessions (90 minutes duration) per week of assisted walking using the exoskeleton technology with elderly females. Spenko et al. [28] investigated the effectiveness of a robotic personal mobility aid with sensors to guide elderly ambulatory individuals away from obstacles. Analysis of the effectiveness of the technology was difficult to interpret as only descriptive data were presented in the paper. Saeki et al. [32] presented a case study describing the use of an upper limb robotic trainer in an elderly woman two years post hemiparesis. Improvements in motor function were reported in musculature of the proximal arm compared to the distal hand and alterations in cortical representation maps of the affected area were suggestive of plastic adaptations. However, these cortical representation changes were not correlated with changes in movement performance of the hemi paretic upper limb. Finally, a recent study by Carlson and Demiris [51] demonstrated improvements in wheelchair mobility when combined with a robotic interface (collaborative control) compared to when participants had to control the wheelchair manually without robotic assistance. Moreover these authors showed, via self-reported questionnaire, that participants found manoeuvering the wheelchair less mentally demanding during collaborative control. 

## 4. Discussion 

This systematic review has highlighted that robotics is still an emerging field in terms of its application to health and rehabilitation for community dwelling older people. Despite these studies being of a lower design quality, the evidence to date shows that robotics research is used widely in engineering laboratories and, to a lesser extent, in clinical settings. Only a very small number of controlled clinical trials evaluated the effects of implementing robotic technology in the home for the purposes of potentially assisting with daily living activities, home care, home maintenance and housework, security, safety, falls detection, or social interaction. Moreover, none of the studies on robotics presented costing of the devices, discussed safety concerns to the user, whether the devices could be mass produced, or social issues such as acceptance by older people in their home environment. 

It was also notable that the studies in this paper focussed on application of robotic technologies for purposes of movement rehabilitation in people who had impairments and disabilities arising from conditions such as arthritis, back pain, balance impairment, stroke, or spinal cord injury. To date no studies have objectively measured the potential application of robotic technologies as monitoring devices in the home setting. Potentially artificial intelligence could be used to measure the health status of their “owner,” provide reminders for specific medications to be taken; or provide contingency procedures in the case of an adverse event such as a slip, trip, or fall.

One study in this paper demonstrated an increased exercise capacity when healthy older participants utilised a robotic exoskeleton for walking training in a “home” setting [40]; however as the study was limited to only one group, with no direct comparison to an age-matched control group who participated in the walking program without the exoskeleton, it is difficult to rigorously evaluate the effectiveness of the use of the robotic exoskeleton in this study. Moreover, follow-up data measures were not taken, therefore it is not possible to ascertain the long-term effectiveness of the technology in assisting in maintaining independence.

However, this paper has demonstrated that applications of robotic technologies have progressed much further than what the general public perceive robots are capable of undertaking. Robotic technology studies, despite being methodologically weak [52], have demonstrated capability of functional improvements following loss of function in upper and lower limbs, or to assist with mobility in indoor environments. The range of the robotic technologies presented in in Table 2 show that the technology is now progressing to the point that that home trials of these different robotic technologies will be undertaken in the near future. 

A limitation of this review was that non-English language studies were excluded. Therefore it is possible that studies of testing robotics in the home environment have been completed, but were not included in this paper as they were published in languages other than English. A second limitation of this review was the decision by the authors to exclude robotic interventions for uses relating to cognitive decline/successful brain aging. Indeed, recent reviews have discussed the use of robotics for cognitive healthcare in the elderly [3]; however, the primary aim of this paper was to review evidence for robotics in addressing physical mobility to reduce disability and loss of independence in the home. Further, although outside the scope of this review and thus also excluded was the emergence of nanotechnology. It is plausible to suggest that progress in nanotechnology research (also known as nanorobotics) [53] could potentially reduce hazards in the home. Robotics will improve, in a number of different directions, to the point of assisting older people to live independently and safely in their homes, and enjoy excellent quality of life in their communities.

The recently released (April 2012) Living Longer. Living Better Report from the Australian Federal Government [54] in response to the Productivity Commission's Report on Care of Older Australians [55] recommends the major expansion of home care supportive services, although these are largely conceptualized as intensive case management services. However, the aged housing and care industry in Australia is moving ahead with the rapid take up of new technologies to assist older people to live more independently at home and in supported accommodation in association with the rollout of a new broadband network nationally [55]. 

Robotics are perhaps one of the newest technology areas to have entered the home care market, being previously largely developed for application in heavy industry and acute health. Looking forward, however, the potential for robotic application in the home is wide open. Some of the major barriers relate to cost of development, the incorporation of artificial intelligence in new design applications, and the encouragement of greater interdisciplinary convergence between the many research fields now involved in the development of new robotic technologies. At this point in time in Australia, progress on home grown robotic applications is limited, given the substantial infrastructure required in the start-up phase [56].

In light of the research reviewed, a number of key recommendations can be provided as follows:

### 4.1. Applying Research into Home Environments

The evidence from the current systematic review has clearly demonstrated that robotics research needs to be conducted in the home environment. To date, only four studies have attempted to conduct research within the home environment [28, 32, 40, 51]. These studies have demonstrated positive outcomes, providing a good rationale to take robotics into environments outside of laboratories or hospital clinics.

### 4.2. Diversifying Robotics

The majority of robotic technology studies found in the current review were directed at movement rehabilitation. However, for the elderly population, healthy living includes prompting and reminding for effective monitoring. Development of robotic technologies should include technologies that can provide gentle reminders for medications, continually scan the environment to ensure no falls have taken place, and have a protocol in place to advise relevant authorities if an incident has occurred. Similarly, robotics has the potential to allow for social connectedness by providing company for elderly people living alone, or to serve as interfaces for connecting with family and friends using existing technologies (e.g., Skype).

### 4.3. Reducing Costs

The final recommendation would be to investigate ways of reducing the costs of technologies. As shown in this paper, robotic technologies are still in development and trial phases. It would be anticipated that with commercial development and mass production, these costs would reduce significantly. However, at the present time costs appear to be a barrier towards broad adoption of robots in the home environment.

In conclusion, this systematic review has shown that robotic technologies have the potential to assist older people and people with disabilities to remain mobile and to live safe and healthy lives at home. Further research and training of the heath and disability workforces is needed to the adoption of robotics as an effective, routine, and practical option within the home environment. The evidence demonstrates that robots already exist to assist with movements, obstacle-avoidance, and functional rehabilitation, but require further development to realise their full potential for safety monitoring, falls, and social connectedness. Future robot design needs to consider development from a different perspective, considering not only assisted mobility, but also interfacing artificial intelligence for interaction with older individuals, to monitor their health, provide medication prompts, encourage exercise, and provide them with confidence to maintain independent living.

## Figures and Tables

**Table 1:** Studies for question 1.

First Author/year	Study Design	Evidence Level [16]	Duration	Setting	Country	Sample size	Age mean	Age range	Characteristics	Gender		Sampling	Technology	Description
										M	F			
Aisen [17] 1997	Intervention study	III-1	Overall duration not stated. Robot trained 4-5 hrs/wk on top of conventional training. Sham trained 1-2 sessions/wk	Hospital rehabilitation clinic	USA	20	§	Robot trained—45–68; Sham trained—38–72	Post-stroke, hemiplegia	11	9	Pseudorandomised	“MIT-MANUS”	Robotic upper limb exoskeleton.
Morvan [18] 1997	Qualitative study	IV	<1 mnth	§	France	28	§	§	Young with either tetraplegia, myopathies or spasticity	#		Not stated	“MASTER” robotic arm system	Psychological preparedness by older people for robots.
Krebs [19] 1998	Intervention study	III-1	Overall duration not stated. Robot trained 4-5 hrs/wk on top of conventional training. Sham trained 1 sessions/wk	Hospital rehabilitation clinic	USA	20	Robot trained—58.5; Sham trained—63	§	§	#		Pseudorandomised	“MIT-MANUS”	Robotic upper limb exoskeleton.
Cozens [20]1999	Intervention study	III-3	<1 d	Laboratory	England	10	§	47–69	Stroke or MS with upper limb weakness	#		Pseudorandomised	No name provided	Robotic upper limb apparatus.
Volpe [21] 1999	Intervention study	III-1	1 wk treatment, 3 yr follow-up	Hospital rehabilitation clinic	USA	20 total, 12 of 20 measureat 3 yrs	Robot trained—54 ± 3 Sham trained—66 ± 2	§	Post-stroke	7	5	Pseudorandomised	“MIT-MANUS”	Robotic upper limb exoskeleton.
Reinkensmeyer [22] 1999	Intervention study	III-3	<1 d	§	USA	5	§	24–79	Brain injury (TBA/ABI)	#		Convenience	Robotic arm	Arm guidance system.
Burgar[23] 2000	Intervention study (x3)	III-2	1 wk–2 mnths	Laboratory	USA	24	§	21–80	Post-stroke hemiplegia	#		Convenience	MIME	Mirror Image Motion Enabler (MIME).
Volpe [24] 2000	Intervention study	III-1	25 × 1 hr sessions 5 d/wk	Hospital rehabilitation clinic	USA	56	64.5	27–83	Post-stroke hemiplegia	30	26	Randomised control	“MIT-MANUS”	Robotic upper limb exoskeleton.
Jezernik [25] 2003	Intervention study	III-3	2 × 1 hr sessions	Spinal cord injury clinic	Switzerland	6	§	38–73	Spinal cord	#		§	“Lokomat”	Robotic gait exoskeleton.
Loureiro [26] 2003	Intervention study	III-3	9 sessions over 3 wks	Hospital	England	30	§	§	Stroke hemiplegia	#		Randomised control	“GENTLE/S”	Haptic upper limb system.
Rentschler [13] 2003	Technical report	IV	<1 d	Laboratory	USA	1	29	29	Healthy	1	—	Case study	PAMA	Personal adaptive mobility aid (PAMA).
Winchester [27] 2005	Other	III-3	12 wks	Laboratory	USA	4	§	20–49	Spinal cord injury	4	—	Convenience	“Lokomat”	Robotic gait exoskeleton.
Spenko [28] 2006	Other	III-3	<1 d	Laboratory	USA	6	§	85–95	Healthy older	1	5	Convenience	“Smartcane” and “Smart walker”	Walking aid for mobility and monitoring.
Isreal[29] 2006	Other	III-3	5 sessions	Laboratory	USA	12	§	15–59	Spinal cord injury	#		Convenience	“Lokomat”	Robotic gait exoskeleton.
Mehrholz[30] 2007	Systematic review	I	n/a	n/a	n/a	n/a	n/a	n/a	n/a	n/a		n/a	Assisted gait device	Robotic-assisted gait training.
Rocon [31] 2007	Other	III-3	<1 d	Laboratory	Spain	10	52.3	§	Tremor	7	3	Convenience	“WOTAS”	Robotic exoskeleton to reduce arm tremor.
Saeki [32] 2008	Other	IV	6 mnths	Laboratory	Japan	1	48	n/a	Neuro-logical	—	1	N/a	“Bi-Manu-Track”	Robotic arm trainer.
Hidler [33] 2008	Intervention study	III-2	6 mnths	Laboratory	USA	5	44.1	24–59	Spinal cord injury	#		Randomised control	“Lokomat”	Robotic gait exoskeleton.
Janssen and Pringle [34] 2008	Intervention study	III-3	6 wks	Laboratory	USA	12	36	20–70	Spinal cord injury	12	—	Convenience	“ERGYS 1”	Functional electrical stimulator leg ergometry.
Krebs [35] 2008	Intervention study	III-2	6 wks	Rehabilitation clinic	USA	47	57.5	27–79	Stroke	#		Pre-post single group	“MIT-MANUS”	Robotic hand visuomotorguidancesystem.
Patton [36] 2008	Other	n/a	n/a	n/a	USA	n/a	n/a	n/a	n/a	n/a		n/a	“KineAssist”	Discussion paper on robot to improve balance and gait.
Querry [37] 2008	Intervention study	III-2	<1 d	Laboratory	USA	26	35.5	§	Spinal cord injury	17	9	Non-randomised control	“Lokomat”	Robotic gait exoskeleton.
Rentschler [38] 2008	Intervention study	III-2	1 d	Laboratory	USA	17	85.3	§	Healthy	#		Pseudorandomised	“GUIDO”	Robotic walker.
Galluppi [39] 2009	Intervention study	IV	§	Hospital	Italy	§	§	§	§	#		§	Robotic wheelchair	Collaborative control robotic wheelchair.
Shimada [40] 2009	Intervention study	III-2	<6 mths	Retirement village	Japan	15	78.3	72–85	Healthy	0	15	Convenience	Stride assistance system	Robotic exoskeleton stride assistance system to assist with walking but provide resistance for physical improvement.
Flinn [41] 2009	Case study	IV	6 wks	Hospital	USA	1	48	n/a	Post-stroke	n/a		n/a	“InMotion2”	Upper limb visuomotor guidance system.
Zeng [42] 2009	Intervention study	IV	§	Hospital rehabilitation clinic	Singapore	3	§	16–48	Cerebral palsy/TBI	#		Convenience	Robotic wheelchair	Collaborative control robotic wheelchair.
Lo [43] 2010	Intervention study	II	12 weeks (total of 36 hours training)	Multi-rehabilitation centres	USA	127	64.6	§	>6 months post-stroke	122	5	Random control trial	Modular robotic system (no name) for upper arm guidance.	Modular robotic upper arm guidance system for shoulder, forearm, wrist, and grasping movements.
Frizera Neto[44] 2010	Intervention study	III-3	<1 d	Indoor installation	Spain	5	§	§	Healthy	#		Convenience	“SIMBIOSIS”	Robotic walker—upper body force interaction.
Sharma [45] 2010	Intervention study	III-3	<1 d	Laboratory	USA	19	38.5	§	Healthy	13	6	Convenience	“Drive Safe” smart wheelchairs	Joystick driven, sensor controlled wheelchairs.
Wolpaw [46] 2010	Expert opinion	n/a	n/a	n/a	n/a	n/a	n/a	n/a	n/a	n/a		n/a	Brain-computer interfaces	Opinion based article on the progression in brain-computer interfaces and suggestions on where the technology paradigm should progress.
Galvez [47] 2011	Intervention study	III-3	n/a	Laboratory	USA	4	§	24–62	Spinal cord injury	#		Convenience	Sensor orthoses	Robotic body-weight support treadmill.
Turiel [48] 2011	Intervention study	III-3	1 hr/d, 5 d/wk, 30–45 mins/session	Laboratory	Italy	14	50.6	n/a	Spinal cord injury	10	4	Pre-post single group	“Lokomat”	Robotic gait exoskeleton.
Schwartz [49] 2011	Intervention study	III-3	2-3 times/wk, 30–45 mins/session	Rehabilitaion clinic	Israel	28	42	n/a	Spinal cord injury	18	10	Single group, matched historical control	“Lokomat”	Robotic gait exoskeleton.
Conroy[50] 2011	Intervention study	II	60 mins, 3 times/wk for 6 wks	Laboratory	USA	62	57.8	n/a	Stroke, hemiplegia upper limb	34	28	Random control trial	“InMotion2”	Upper limb visuomotor guidance system. 2D versus 3D including antigravity training, comparing the combination of vertical and planar robot with planar alone.
Carlson and Demiris[51] 2012	Intervention study	III-2	<1 d, 2 × 40 min sessions	Simulated home	England	21	§	17–47	Healthy	#		Convenience	No name	Collaborative controlled robotic wheelchair.

^#^No gender given, ^§^Not given.

**Table 2:** Quality evaluation of data driven studies for question 2.

First Author/year	Design	Evidence Level [16]	Duration	Dosage	Setting	Country	Sample size	Age mean	Age range	Characteristics	Gender		Sampling	Key DV	Key measure	Results	Score (max27)* [15]
											M	F					
Spenko [28] 2006	Pilot Intervention study	IV	§	§	Residential care	USA	“several”	§	§	§	§	§	§	Distance from wall in meters before the smart-walker changed direction	Motor performance	Good performance outcome using both PAMM technologies. Subjective measures gave less confidence in robotic-controlled walkers to manually controlled walkers.	4
Saeki [32] 2008	Case study	IV	<6 mths	20 mins 2 d/wk for 4 mths	Home	Japan	1	48	§	Neurological	0	1	§	Oxygenated/ deoxygenated/ total haemoglobin level in motor cortex assessed by fMRI technique	Motor assessment scale (modified Ashworth Scale)	Improvement in modified Ashworth motor assessment scale (2 to 5); reduced score on modified Ashworth scale (3 to 2); no change in wrist and fine motor tasks; direct activation of motor area in affected hemisphere.	4
Shimada [40] 2009	Intervention study	III-2	<6 mths	2 × 90 mins/wk for 3 mths	Retirement village	Japan	15	78.3	72–85	Healthy	0	15	Conven-ience	Walking speed	5 m walk test	Increase in walking speed; reduction in energy consumption (lower glucose metabolism)	12
Carlson and Demiris [51] 2012	Intervention study	III-2	<1 d	2 × 40 mins	Simulated “home” environment	England	21	§	17–47	Healthy	§	§	Conven-ience	Wheel-chair control around a simulated home environment	Collision and cognitive perception	Less collisions with robotic assistance and lower scores on perceptions of concentration	13

^§^Not given.

*The last question of the Downs and Black assessment tool was excluded due to ambiguity of the question. This meant that the maximum score possible was 27.
